# Lack of Guanylate Cyclase C results in increased mortality in mice following liver injury

**DOI:** 10.1186/1471-230X-10-86

**Published:** 2010-08-02

**Authors:** Elizabeth A Mann, Kumar Shanmukhappa, Mitchell B Cohen

**Affiliations:** 1 Division of Gastroenterology, Hepatology, and Nutrition, Cincinnati Children's Hospital Medical Center and the University of Cincinnati, Cincinnati, OH, USA; 2 Current address: Section of Comparative Pathology, New England Primate Research Center, Harvard Medical School, Southborough, MA, USA

## Abstract

**Background:**

Guanylate Cyclase C (GC-C) expression in the intestine plays a role in the regulation of fluid and ion transport, as well as epithelial cell apoptosis and proliferation. In the adult rat liver, GC-C expression is increased in response to injury. We hypothesized that GC-C is required for repair/recovery from liver injury.

**Methods:**

We subjected wild type (WT) and GC-C deficient mice to acute liver injury with a single injection of the hepatotoxin carbon tetrachloride. Changes in the level of expression of GC-C and its ligands uroguanylin and guanylin were quantified by real-time PCR. Liver morphology, and hepatocyte necrosis, apoptosis and proliferation, were examined at 1-3 days post-injury in mice on a mixed genetic background. Survival was followed for 14 days after carbon tetrachloride injection in wild type and GC-C deficient mice on both a mixed genetic background and on an inbred C57BL6/J background.

**Results:**

GC-C deficient mice on the mixed genetic background nearly all died (median survival of 5 days) following carbon tetrachloride injection while WT littermates experienced only 35% mortality. Elevated levels of TUNEL-positive hepatocyte death on post-injury day 1, increased apoptosis on day 2, and increased areas of centrilobular necrosis on days 2 and 3, were evident in livers from GC-C null mice compared to WT. Collectively these data suggest increased hepatocyte death in the GC-C null mice in the early time period after injury. This corresponds temporally with increased expression of GC-C and its ligands guanylin and uroguanylin in post-injury WT mouse liver. The hepatocyte proliferative response to injury was the same in both genotypes. In contrast, there was no difference in survival between GC-C null and WT mice on the inbred C57BL/6 J background in response to acute liver injury.

**Conclusions:**

Signalling via GC-C promotes hepatocyte survival *in vivo *and is required for effective recovery from acute toxic injury to the liver in a strain-specific manner.

## Background

The family of particulate guanylate cyclases (GC) is known to play substantial roles in the function of a number of different organs. These membrane receptors include the natriuretic peptide receptors GC-A and GC-B in the cardiovascular system, and GC-E and GC-F in the retinal phototransduction system (reviewed in [1]). GC-C is most highly expressed in the intestine where it is the receptor for the peptides guanylin (Gn)[2] and uroguanylin (Ugn)[3] and contributes to the maintenance of fluid and salt homeostasis via ligand-activated cGMP production. Activation of this receptor by the bacterial heat-stable enterotoxin[4], which is very similar in sequence and structure to the mammalian peptides Gn and Ugn, results in secretory diarrhea in both animals and man and is a major health problem. Recent advances in our understanding of GC-C mediated signaling in intestinal epithelial cells have suggested a role in basic cellular processes including apoptosis and proliferation ([5-9].

While highest in the intestine, GC-C expression is also found in other tissues including kidney[10], pancreas[11], and liver. Similar to its expression in the intestine, GC-C is temporally regulated in the liver of rodents and is expressed at highest levels in the perinatal period [12,13]. In the adult liver, GC-C expression is undetectable (by Northern analysis) but is up-regulated in injury/regeneration models, including exposure to the hepatotoxin CCl_4 _and by partial hepatectomy [14,15]. In mice with a deficiency of GC-C the liver appears normal and no phenotype has been described under specific-pathogen free husbandry[16,17].

Administration of CCl4 is a widely used model of necroinflammatory liver injury and regeneration. In centrilobular hepatocytes of the liver, cytochrome P450 enzymes mediate metabolism of CCl_4 _into toxic free radicals which cause lipid peroxidation and membrane damage, eventually resulting in necrosis [18]. Hepatocyte death due to apoptosis via activation of caspase 3, although not as prominent as death due to necrosis, has also been demonstrated to play a role in CCl_4 _injury [19-22]. Proliferation of surviving hepatocytes, along with removal of cellular debris and restoration of the extracellular matrix, ultimately results in liver regeneration [23]. In order to explore the role of GC-C in the liver, we compared the response of wild type (WT) and GC-C null mice to acute CCl_4 _injury.

## Methods

### Mice

GC-C knock out (KO) mice with a targeted disruption of the *Gucy2c *gene [16] were maintained on a C57BL/6J background, following 10 backcross generations. Where indicated, these G-CC null mice were crossed with Black Swiss outbred mice (NTac:NIHBS, Taconic, Hudson, NY) and the resulting heterozygous mice mated to generate homozygous wild type (WT) controls and GC-C null littermates (F2 generation). All mice were genotyped by PCR. Mice were housed under specific-pathogen free conditions and fed food and water *ad libitum*. Adult mice of both sexes, aged 8-12 weeks, were used in experiments. Animal protocols were approved by the Institutional Animal Care and Use Committee of the Cincinnati Children's Hospital Medical Center (Cincinnati, OH).

### Acute Liver Injury

Mice were exposed to a single intraperitoneal injection of CCl_4 _(Sigma-Aldrich, St. Louis, MO) at a dose of 1 μl CCl_4 _per gram body weight as a 50% solution in corn oil. Vehicle control mice were injected with corn oil alone. Injections were performed within a 2 hr window to minimize any variation due to circadian rhythm. Mice were examined daily and sacrificed at the indicated times. The liver was excised, weighed, and divided into aliquots which were snap-frozen for later analysis, or fixed in formalin. After paraffin embedding, sections were either stained with hematoxylin and eosin for light microscopic analysis or reserved for immunohistochemistry.

### Hepatocyte Death

DNA fragmentation was examined using TUNEL staining as described [5]. Briefly, tissue sections were digested with Proteinase K, and then incubated in freshly made reaction buffer containing terminal transferase and biotin-16-dUTP (Roche Diagnostic, Indianapolis, IN) to end-label DNA fragments. Labeled DNA was detected using the Vectastain ABC-alkaline phosphatase system (Vector Laboratories, Burlingame, CA) and Sigma Fast Red (Sigma-Aldrich). As a negative control, terminal transferase was omitted from the reaction buffer. To measure hepatocyte death at 24 hours post- CCl_4 _injection, the number of labeled nuclei present in 3 high-power fields per liver was counted by an investigator unaware of the mouse genotype. Intense staining of necrotic areas at later time points prevented similar analyses. Instead, the area of necrotic damage compared to the total area of the field, excluding blood vessels and ducts, was determined using ImageJ version 1.38 (National Institutes of Health, Bethesda, MD), and a percentage of necrotic damage was calculated for each liver sample.

### Apoptosis Detection

Paraffin-embedded liver sections were stained with antibody against activated caspase 3 (1:250 dilution, Cleaved Caspase 3 (Asp175) Antibody, Cell Signaling Technology, Inc., Danvers, MA) and counterstained with hematoxylin as described [5]. To quantify, cleaved caspase 3 -positive cells were counted in 10 randomly selected fields per mouse liver (200× magnification) by an investigator unaware of the genotype. Caspase 3 activity was measured using a colorimetric substrate (Ac-DEVD-pNA, Enzo Life Sciences, Plymouth Meeting, PA). An aliquot of frozen liver was homogenized in lysis buffer containing 50 mM HEPES, pH 7.4, 5 mM CHAPS, and 5 mM DTT, and centrifuged at 20,000 × *g *for 30 min at 4°C. Cytosolic protein (100 μg as determined by the Bradford assay, Bio-Rad, Hercules, CA) was incubated overnight at 37°C with 20 mM Ac-DEVD-pNA in assay buffer (20 mM HEPES, pH 7.4, 2 mM EDTA, 0.1% CHAPS, and 5 mM DTT). The amount of pNA released from the substrate was measured by determining the absorbance value at 405 nm. The activity in control livers from mice injected with vehicle alone did not differ between genotypes so the combined average value was used for normalization.

### Hepatocyte Proliferation

Proliferating cell nuclear antigen (PCNA) staining was used as a marker for hepatocyte proliferation that occurs in response to CCl_4 _injury. Tissue sections were deparaffinized, rehydrated and endogenous peroxidase activity quenched with 1% hydrogen peroxide for 15 min. After washing, tissues were heated in 0.01 M sodium citrate, pH 6, to promote antigen exposure. Sections were then washed, blocked in 3% normal goat serum, and incubated with PCNA polyclonal antibody (1:500, Santa Cruz Biotechnology, Santa Cruz, CA) overnight at 4°C. Following washes, sections were incubated with biotinylated anti-rabbit secondary antibody and labeled nuclei detected using the Vectastain ABC-horseradish peroxidase kit and 3,3-diaminobenzidine (Vector Laboratories). Sections were counterstained with hematoxylin (Fisher Scientific, Pittsburgh, PA). As a negative control, primary antibody was omitted. For each tissue, the hepatocyte labeling index (percent of nuclei that stain positive for PCNA) was determined. Both PCNA-labeled and unlabeled hepatocyte nuclei were counted in 3 high-power fields (≥ 1100 nuclei per tissue) in a blinded fashion.

### Quantitative Real-Time PCR

Total RNA was isolated from frozen liver using Tri Reagent (Molecular Research Center, Inc., Cincinnati, OH) according to the manufacturer's protocol. RNA samples were treated with DNase I (DNA-*free*, Ambion, Austin, TX) and reverse-transcribed (2 μg) using random decamers (RETROscript, Ambion). PCR reactions using specific gene primers were performed with Brilliant II SYBR Green QPCR mix (Stratagene, La Jolla, CA) in the Mx3000p thermocycler (Stratagene). A relative amount for each gene examined was obtained from a standard curve generated by plotting the cycle threshold value against the concentration of a serially diluted RNA sample expressing the gene of interest. This amount was normalized to the level of 18 S RNA, which did not vary with CCl_4 _treatment. Primer sequences used were as follows: GCC, forward 5'-CGAAAGCGCCTGCGTGAAGC and reverse 5'-TTCACAGGTGCTGCTCCGGC; 18 S, forward 5'-GATCCGAGGGCCTCACTAAAC and reverse 5'-AGTCCCTGCCCTTTGTACACA; Ugn, forward 5'-TGAGTTGGAGGAGAAGGAGATGTC and reverse 5'-AAGGGCAAGGCTGGGTTATG; Gn, forward 5'-GAGTGACATCGCTTGCCTTTC and reverse 5'-TGAGTTTGTTAGCCTCGTGACTTC; and Cyp2e1, forward 5'-TCAAAAAGACCAAAGGCCAGC and reverse 5'-TCCGCAATGACATTGCAGG [24].

### Statistics

Kaplan-Meier survival curves were generated for each genotype and the log-rank test used for comparison (Prism, GraphPad, San Diego, CA). The Mann-Whitney test was used to evaluate the statistical significance between 2 groups (Prism). A *P *value less than or equal to 0.05 was considered significant.

## Results

### Decreased survival of GC-C deficient mice following liver injury is strain-specific

**Figure 1:**
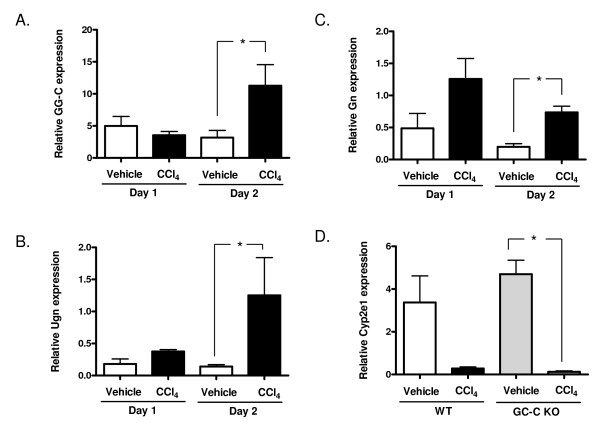
**Expression of GC-C, Ugn and Gn is increased after acute liver injury**. Quantitative real-time PCR was used to examine expression at 1 and 2 days after CCl_4 _injection compared to vehicle (corn oil alone) injected mice. Expression of the receptor (A) and both ligand genes (B and C) is increased at 2 days after CCl_4 _injection. (D) Cyp2e1 expression is decreased in both genotypes after CCl_4 _injection. Similar levels of Cyp2e1 are seen in vehicle injected mice by real-time PCR analysis and are sharply reduced by Day 1. *P = 0.03, N = 3-4 mice per time point.

Our preliminary work [25] suggested a decreased survival in GC-C KO mice on a mixed genetic background (129 and Black Swiss) compared to WT littermates in response to a single intraperitoneal injection of CCl_4_. To recapitulate the mixed genetic background of these findings we bred GC-C KO mice (> 10^th ^generation C57BL/6J backcross) with outbred Black Swiss mice and utilized homozygous WT and GC-C KO littermates (F2 generation). We first determined expression levels of GC-C and its ligands, Gn and Ugn, in livers of WT mice. Quantitative analysis by real-time PCR showed that GC-C expression is relatively low in RNA from control liver (approximately 20 times lower than in ileum, data not shown). Two days after a single injection of CCl_4 _(1 μl CCl_4 _per gram body weight) expression of GC-C in the liver doubled (Figure 1A) compared to vehicle-treated mice (P ≤ 0.03, N = 3-4 mice per time point). Ugn and Gn expression is detectable in control liver by real-time PCR (Figure 1B and 1C). After CCl_4 _administration, the expression of both GC-C ligands also increased significantly (P = 0.03, vehicle Day 2 vs. CCl_4 _Day 2, N = 4 mice per time point).

**Figure 2:**
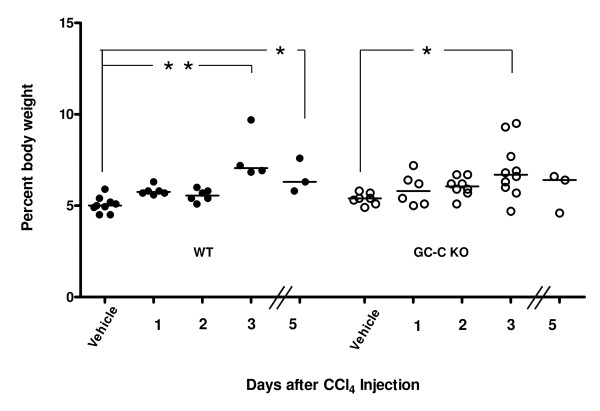
**Increase in liver mass following CCl_4 _treatment**. For both genotypes liver mass (presented as the percent of body weight for each mouse, bar represents the median value) increased after CCl_4 _injury as expected. The difference was statistically significant at both Day 3 and Day 5 for the WT mice (vs. WT vehicle, * *P *= 0.05, ** *P *= 0.01). Liver mass increases in a similar fashion for GC-C KO mice up to Day 3 (GC-C KO vehicle vs. Day 3, * *P *= 0.05) but is not significantly different by Day 5.

**Figure 3:**
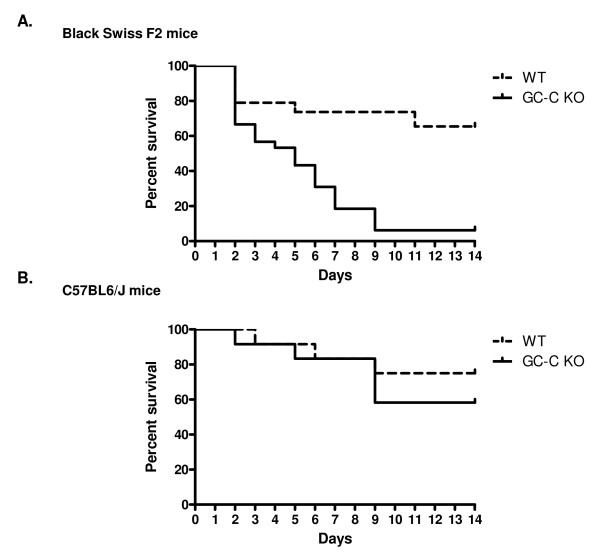
**Survival of GC-C KO mice following acute CCl_4 _injury**. Mice were injected intraperitoneally with 1 μl CCl_4 _per gram body weight as a 50% solution in corn oil and checked daily for 2 weeks. (A) In the outbred Black Swiss F2 generation, 65% of WT littermates survived while only 1 GC-C null mouse was alive at Day 14 post-injection. (*P *= 0.0008, N = 19 WT mice, N = 30 GC-C null mice). (B) No difference in survival is seen between the 2 genotypes in the C57BL/6 J background. N = 12 mice per group.

We next compared the response to CCl_4 _-induced liver injury in WT and GC-C KO littermates. Cyp2e1 is a key cytochrome P450 enzyme responsible for metabolism of CCl_4 _into hepatotoxic radicals. Expression levels in the liver were similar between vehicle injected WT and GC-C KO mice and decreased markedly in both at Day 1 (Figure 1D) as has been previously described in the CCl_4 _model [24]. As shown in Figure 2, the liver-to-body weight ratio, which is reflective of liver regeneration [26,27] increased as expected after CCl_4 _injection in WT mice and was significantly different from control at both Days 3 and 5. Liver mass increased in a similar fashion for GC-C KO mice up to Day 3 (GC-C KO vehicle, n = 7 vs. Day 3, n = 10, * *P *= 0.05) but was not significantly different at Day 5 (n = 3). By Day 5 it was obvious that there was more lethality in GC-C KO mice compared to WT in response to CCl_4 _administration. Additional mice of both genotypes were injected and survival was documented for 14 days. WT littermates exhibited 65% survival through Day 14 (Figure 3A) while nearly all GC-C null mice died by Day 9 following injection (*P *= 0.001, median survival 5 days), thus indicating a protective role for GC-C action throughout this time period in mice of mixed genetic background.

We asked whether GC-C signaling was also a factor in survival of mice on the inbred C57BL/6 background (> 10 backcross generations). As shown in Figure 1B, there was no significant difference in survival between GC-C null and WT mice (58% vs. 75%) on the inbred background. Therefore, the protective role of GC-C in liver injury is strain-specific. We utilized mice of mixed genetic background to further characterize the role of GC-C in our subsequent studies since they demonstrated a profound difference in survival.

### Comparison of hepatocyte death in WT and GC-C null mice

**Figure 4:**
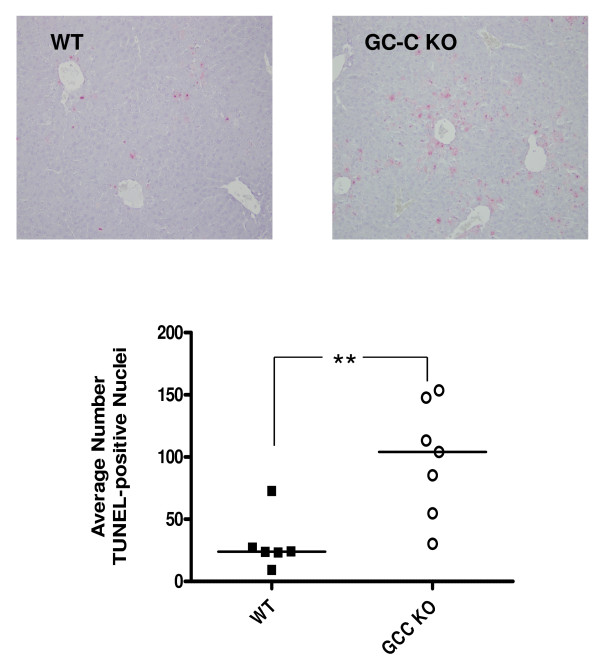
**Enhanced hepatocyte damage in GC-C null mice after CCl_4 _injection**. TUNEL staining was used to examine the level of DNA fragmentation in hepatocyte nuclei in liver sections from Day 1 treated mice. *Top*. Representative photomicrographs are shown (magnification 200×). *Bottom*. The number of TUNEL-labeled nuclei was counted in 3 fields (magnification 200×) for each mouse and presented as the average. The median number of TUNEL-positive nuclei in GC-C KO liver was 4 times that in WT liver (**P = 0.005; N = 6-7 mice per group).

**Figure 5:**
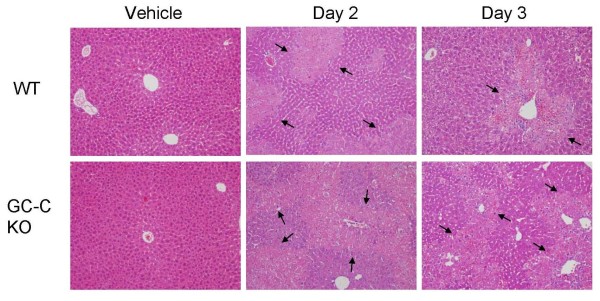
**Histological analysis of WT and GC-C null mouse livers after CCl_4 _treatment**. Paraffin-embedded sections from livers obtained 2 and 3 days after injection of 1 μl CCl_4 _per gram body weight in corn oil or corn oil only (vehicle) were stained with H&E. All CCl_4 _treated sections show similar features of centrilobular necrosis as indicated by arrows, which appears to be more extensive in the GC-C KO (see Figure 6 for quantification). Magnification 200×.

**Figure 6:**
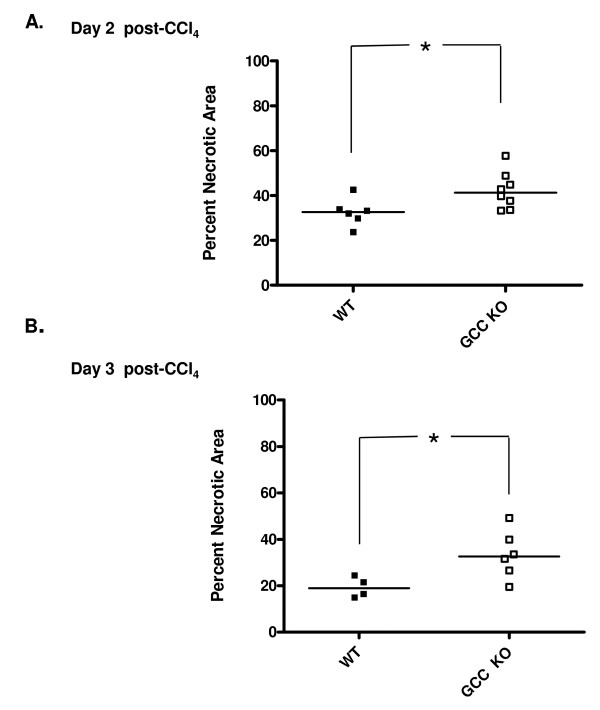
**Increased area of necrotic damage in GC-C null mice**. H&E stained liver sections were evaluated by morphometric image analysis to determine the extent of necrotic area. While the range overlaps, GC-C KO mice exhibited more necrotic liver damage at both 2 days (A) and 3 days (B) after CCl_4 _injection (*P = 0.04, N = 4-8 mice per genotype). Data is presented as the mean percentage necrosis in 3 fields (100×) per mouse. The bar is drawn at the median value for all groups.

In response to CCl_4 _exposure, centrilobular hepatocyte necrosis and apoptosis occur during the injury process. We therefore determined the amount of DNA breaks, as assessed by TUNEL staining (Figure 4), in liver sections of both genotypes at 1 Day after CCl_4 _injection. TUNEL-stained nuclei were observed in GC-C KO liver sections at 4 times the level seen in WT liver sections (median KO 104 vs. WT 24 nuclei, *P *= 0.005, N = 6-7 mice per genotype). We next examined H&E stained liver tissue sections from Days 2 and 3 following CCl_4 _injection. The extent of centrilobular necrosis appears to be increased at Days 2 and 3 in the GC-C KO (representative photomicrographs shown in Figure 5) and we used morphometric analysis to determine the extent of necrotic area. The percent necrotic area per liver was estimated from measurements of 3 microscopic fields (100×) per mouse. On both Day 2 and Day 3 (Figure 6) the median extent of necrosis was greater in livers from GC-C KO mice than in WT liver (Day 2, 42% vs. 32% and Day 3, 33% vs.19%, *P *= 0.04, N = 4-8 mice per genotype. By Day 5 all WT mice (N = 3) are in recovery and showed comparable levels of inflammatory infiltrate; however, 1/3 surviving GC-C null mice continued to exhibit some areas of focal necrosis (data not shown).

**Figure 7:**
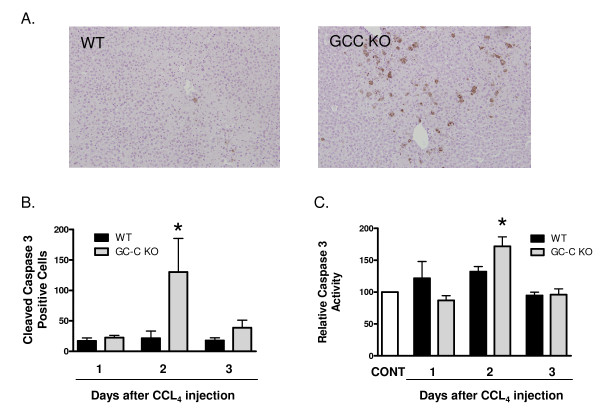
**Increased post-CCl_4 _hepatocyte apoptosis in GC-C null livers **. (A) Representative photomicrographs of cleaved Caspase 3 antibody staining (brown color) in livers (counterstained with hematoxylin) from WT and GC-C KO mice at 2 days after CCl_4 _administration (200 × magnification). (B) Cleaved Caspase 3 stained cells were counted in 10 fields (200×) per mouse at the indicated times. Data is presented as the mean number ± SEM, N = ≥ 4 mice per time point. *P = 0.04, GC-C KO vs. WT, Day 2. (C) Caspase 3 activity was measured using the colorimetric substrate Ac-DEVD-pNA (N = ≥ 3mice per time point). Activity levels were normalized to the level seen in vehicle injected liver (CONT). *P = 0.03, GC-C KO vs. WT, Day 2.

Both necrotic and apoptotic cell death processes can result in TUNEL-positive staining. To look specifically at hepatocyte death caused by apoptosis, we examined liver sections for activated (cleaved) caspase 3 staining by immunohistochemistry (IHC.) No staining was detected in any of the vehicle injected control livers (not shown). A low level of cleaved caspase 3-stained hepatocytes was visible in sections from WT CCl_4 _-injected mice (Figure 7A). Stained cells were detectable at equivalent levels in WT mice from Day 1 through Day 3 post-CCl_4 _(Figure 7B). In contrast, by Day 2 the number of stained cells was significantly greater in GC-C KO livers (*P *= 0.04), and then decreased by Day 3 to levels comparable to WT. To confirm these findings we also determined Caspase 3 activity in liver homogenates using a colorimetric substrate (Figure 7C). Similar to our IHC findings, the level of Caspase 3 activity was greater in GC-C KO livers than WT at Day 2 (*P *= 0.03).

### Hepatocyte proliferation is normal in GC-C null mice

**Figure 8:**
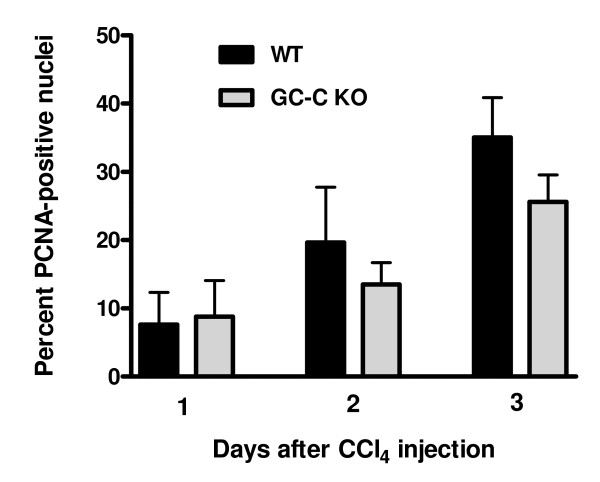
**Timing and extent of hepatocyte proliferation post-CCl_4 _is similar in both GC-C KO and WT**. PCNA IHC was used to examine hepatocyte entry into the cell cycle. Both PCNA-labeled and unlabeled nuclei (counterstained with hematoxylin) were counted in 3 fields (200×) and the percentage of PCNA-positive nuclei was calculated (≥ 1100 total nuclei per mouse, N = ≥ 4 mice per time point).

We next examined the timing and extent of the wave of hepatocyte proliferation that occurs in response to CCl_4 _-mediated hepatocellular death. PCNA (proliferating cell nuclear antigen) staining is a marker for cellular proliferation and is seen only in nuclei during either G1 or S phases of the cell cycle. As shown in Figure 8, the percent of hepatocyte nuclei positive for PCNA increased in both genotypes from 1 day through 3 days post-CCl_4 _injury. Although there was a trend towards a reduction in the level of PCNA-positive nuclei in the GC-C KO mice, there was no significant difference between the 2 genotypes at any of the time points examined.

## Discussion

This study demonstrates that GC-C, known primarily for its role in intestinal epithelial cell function, also plays an important role in the response to liver injury. GC-C null mice do not survive the acute liver injury mediated by a dose of CCl_4 _that causes 35% lethality in WT mice. The median length of survival is 5 days with almost all GC-C KO mice succumbing by post-injury day 9.

There was no effect on survival in GC-C deficient mice on the C57Bl/6J inbred background following toxic liver injury (Figure 3). Strain-specific differences in the phenotype of gene mutations are found in the literature, especially in the study of multigenic diseases such as cancer and diabetes [28]. For example, the C57BL/6J strain is known to be resistant to liver tumorigenesis in response to the chemical diethylnitrosamine and thus was used to elucidate the role of p27Kip1 as a tumor suppressor [29]. This strain is also resistant to liver fibrosis following chronic CCl_4 _administration, most probably due to a predominantly Th1 cytokine response compared to a Th2 response in susceptible strains [30]. At this time the C57BL/6J-specific factors that are responsible for the successful recovery of GC-C KO mice after liver injury have not been identified. Our use of the outbred Black Swiss mouse was crucial in uncovering the lack of survival following liver injury in the GC-C KO [24] that was not seen on the C57Bl/6J inbred background. However, the concomitant individual variability also complicates the study of the mechanism (s) of GC-C action. We focused our research on comparing the early hallmarks of hepatocyte apoptosis/necrosis and subsequent proliferation in both genotypes. Possible causes of deaths occurring later in the regeneration process have not been explored.

GC-C has been localized to non-parenchymal cells and hepatocytes [15,31] and although the cellular localization of Gn and Ugn is not known their detection and increased expression following CCl_4 _exposure (Figure 1) is consistent with a role for the GC-C receptor signalling system during liver repair. In addition, both Gn [32,33]and Ugn [34,35]are known to circulate in the plasma. In response to CCl_4 _injury GC-C expression is also up-regulated in the mouse liver, as has previously been shown in rats [14]. In primary rat hepatocyte cultures, the addition of glucocorticoids and IL-6 [31], and both insulin and heregulin-β1[36], have been shown to increase GC-C levels. Therefore, we speculate that IL-6 [19], a key coordinator in the response to liver injury, plays a role in mediating the increased expression of GC-C that we and others have observed.

While the level of hepatocyte proliferation after injury is comparable to WT (Figure 8), GC-C deficiency results in increased hepatocyte cell death (4 times the level in WT liver) 1 day after CCl_4 _administration, as well as an increased extent of necrotic areas at later time points (Figures 4 &6). Our Caspase 3 studies (Figure 7) demonstrate that increased apoptosis also contributes to the overall increased hepatocyte death in GC-C null mice. This suggests that the mortality that occurs within a few days of injury may be due to an imbalance in the rates of hepatocyte loss and repopulation. Increased levels of apoptosis have been demonstrated in GC-C KO intestine in a non-lethal radiation model and appear to be mediated by a cGMP-dependent mechanism [5] while one study also demonstrated an increase in crypt cell apoptosis in the GC-C KO intestine at baseline compared to WT mice [6]. It is not known if the protective mechanisms of GC-C signalling in the intestine and liver are similar and further exploration is warranted.

## Conclusions

Lack of GC-C expression in outbred mice versus inbred C57BL6/J mice revealed a strain-specific protective role for GC-C in the liver following acute CCl_4 _injury. In contrast to WT littermates, GC-C null mice exhibited near 100% lethality. Increased hepatocyte cell death due to both necrosis and apoptosis occurred in the days immediately following exposure in the GC-C KO mice and likely contributed to mortality.

## Competing interests

The authors declare that they have no competing interests.

## Authors' contributions

EAM designed and performed the experiments, analyzed the data, and wrote the paper. KS participated in experimental design and in editing of the paper. MBC designed experimental strategy, interpreted the data, and critically edited the paper. All authors read and approved the final manuscript.

## Pre-publication history

The pre-publication history for this paper can be accessed here:

http://www.biomedcentral.com/1471-230X/10/86/prepub
